# Arthroscopic Bankart repair augmented with glenoid bone dry allograft

**DOI:** 10.1016/j.jseint.2024.09.008

**Published:** 2024-09-24

**Authors:** Thomas Boissinot, Antoine Baltassat, Hugo Barret, Mathieu Girard, Pierre Mansat, Nicolas Bonnevialle

**Affiliations:** a CHU de Toulouse, Place du Dr Baylac, Toulouse, France; b Clinique Universitaire du Sport, Hôpital Pierre Paul Riquet, Toulouse, France

**Keywords:** Bankart repair, Allograft, Glenoid bone loss, Shoulder instability, Bone block, Cortical button

## Abstract

**Background:**

The recurrence rate of shoulder instability after arthroscopic isolated Bankart repair is up to 25% at long term, especially in case of bipolar bone loss. Bony augmentation with free bone dry allograft would be an option to reconstruct a glenoid subcritical bone defect and to minimize the failure rate.

**Methods:**

This case series included patients with anterior shoulder instability treated by arthroscopic free bone dry allograft (Supercrit, BIOBank, Lieusaint, Ile-de-France, France), fixed with a cortical button as an augmentation of Bankart repair and reviewed with a minimum follow-up of 2 years. Clinical assessment was based on active range of motion, shoulder apprehension test, as well as Walch-Duplay Score, Rowe Score, and Subjective Shoulder Value. Radiological evaluation was based on postoperative and last follow-up computed tomography scan to assess bone block positioning, glenoid enlargement, allograft healing, and/or resorption.

**Results:**

Five patients were included with a mean follow-up of 50 months (range 44-56). None of the patients reported a recurrence, but apprehension test was positive in one. Mean Walch-Duplay Score, Rowe Score, and Subjective Shoulder Value were 88 points (70-11), 93 points (75-100), and 89% (80-95), respectively. No surgical complications were recorded. The mean preoperative anterior glenoid bone loss was 13.8% (7-19). At last follow-up, graft resorption was observed in all patients, reaching 100% of the initial volume in 4 cases.

**Conclusion:**

This study showed satisfactory clinical results of Bankart repair augmented with dry bone allograft in the treatment of anterior shoulder instability with glenoid subcritical bone loss. However, anatomical results were disappointing, with graft resorption that raises the question of going ahead with such a procedure.

Although primary arthroscopic Bankart capsulo-labral repair has shown satisfactory functional outcomes in case of first anterior shoulder dislocation, recent studies have demonstrated a recurrence rate reaching up to 25% in cases of chronic instability with bone defect, off-track Hill Sachs lesion, overhead/contact sport, or shoulder hyperlaxity.^5^^,^^18^^,^^26^

Glenoid bone loss has been highlighted as a major negative impact on shoulder stability in cases of soft tissue procedures. In a cadaveric study, Itoi et al^19^ demonstrated that, biomechanically, the critical glenoid defect size requiring a bone graft was set at 21%. In this situation, a bony reconstruction would be indicated either by coracoid transfer or other type of graft.^22^ However, recent studies described a gray zone called “subcritical” bone loss, ranging from 13% to 17%, which might also have an important part in soft tissue procedure failure.^11^^,^^21^^,^^25^

Coracoid bone block has proven its efficiency in patients with glenoid bone defects, yet some studies report significant complications. In a systematic review of 35 articles and 2560 cases, Cho et al^8^ recorded a 9.4% intraoperative and postoperative complication rate including nerve injury, infection, nonunion, and hardware failure.

Therefore, as an alternative, glenoid reconstruction using a free bone block was suggested to minimize postoperative complications.^15^ Allograft or autograft from numerous locations can be used (iliac crest, distal, clavicle, proximal tibia, femoral head, and femoral condyle), and these are usually either freshly harvested or cryopreserved in glenoid augmentation procedures.^30^

While autogenic bone graft implies longer operating time and donor site morbity (infection, hematoma, and pain), dry allograft offers higher availability and easier conservation properties than fresh frozen bone transplant.^1^^,^^16^

The aim of our study is to evaluate clinical and radiological outcomes of an arthroscopic Bankart repair augmented with a free dry bone allograft for anterior shoulder instability. We hypothesized that this procedure would provide satisfactory clinical outcomes and that the bone graft would compensate for the bone defect.

## Methods

### Study design

This study was approved by the ethical committee of Toulouse University Hospital, and all the patients gave informed consent (No. RnIPH 2023-32).

This study is a case series from a pilot study to evaluate the Supercrit (BIOBank, Lieusaint, Ile-de-France, France) allograft reconstruction of glenoid defects with concomitant Bankart repair. We included patients (1) who complained of chronic anterior shoulder instability (2) and underwent free bone arthroscopic glenoid dry allograft bone block (3) in addition to a Bankart repair. The minimum (4) clinical and radiological follow-up was 2 years.

We excluded patients who required intraoperative conversion to another surgical technique, and patients who underwent a previous shoulder surgery of the involved side.

From our database, 5 patients met the inclusion criteria. None were lost to follow-up.

### Surgical technique and rehabilitation protocol

All the procedures were performed by the same fellowship-trained surgeon (N. B.).

#### Patient positioning

The patient was positioned in a beach chair under general anesthesia and interscalene nerve block. The upper limb was held with a mobile arm positioner (Trimano Fortis Support Arm; Maquet, Rastatt, Germany).

Preoperative antibiotic prophylaxis was administered 30 minutes before performing surgery. A 1-g tranexamic acid intravenous injection was given prior to skin incision to decrease intraoperative bleeding.

#### Rotator interval opening and glenoid preparation

A 70° arthroscope was used to have a proper view of the anterior aspect of the glenoid throughout the procedure.

First, a standard arthroscopic diagnosis was performed through the posterior standard portal. The glenoid and humeral bone loss were assessed.

The anterior labrum was mobilized from 1 to 6 o’clock. At this point, a stick was used through the anterolateral portal to retract the capsulo-labral complex from the glenoid to create an anterior chamber to easily prepare the glenoid surface.

A motorized surgical rasp was then used to decorticate and flatten the anterior/inferior part of the glenoid neck, to achieve an optimal surface for healing.

#### Glenoid drilling

The scope was placed through the anterior portal. The dedicated glenoid guide (Latarjet Guiding System; Smith & Nephew, Andover, MA, USA) was introduced through the posterior portal. Its hook was slid inside the joint, flush to the glenoid surface, and positioned at 4 o’clock.

The 2.8 mm cannulated glenoid drill was driven through the guide and the glenoid neck, from posterior to anterior. A polydioxanone suture (PDS) was inserted through the drill.

#### Bone graft preparation

A 20 × 10 × 10 mm dry cortico-cancellous bone block (Supercrit) was prepared on the back table. The preloaded device (Osteo-connect; Smith & Nephew, Andover, MA, USA) was placed at the middle of the length at 5 mm width. The peg button was on the cortex of the graft, leaving the under cancellous surface free for glenoid fixation.

#### Bone graft transfer and fixation

The scope was then switched back from anterior to posterior. A 15-mm cannula was placed through the rotator interval from the anterior portal. The PDS suture’s anterior end was used as a shuttle for the cortical button’s strands through the cannula and the glenoid neck. The posterior view helped to adjust the final position of the bone block, parallel to the glenoid rim.

A posterior cortical button was slid along the posterior free strands. A suture tensioner was used for tightening at 100 Newton before final locking with a Nice knot.

#### Bankart repair

A PDS suture was passed through the capsule at 4-5 o’clock position to tighten the capsule from south to north. Two to 3 double-loaded anchors (Genesis press FT 1.8 mm; Conmed, Utica, NY, USA) were placed to fix the labrum, and a capsular shift was performed.

#### Postoperative care

An arm sling was worn for 3 weeks. Rehabilitation began the day after surgery, with pendulum exercises and active anterior elevation. External rotation was protected until 6 weeks postoperative. Return to noncontact sports and heavy lifting was authorized at 3 months. Full range of motion and muscle strength recovery were mandatory before returning to high-risk activities.

### Clinical assessment

Preoperative data were collected from the secured medical file. Postoperatively, clinical evaluation was planned at 2 weeks, 3 months, 6 months, and 2 years. Additional evaluation was optional at last follow-up. Active range of motion was measured, and a shoulder apprehension test was performed. A recurrence of dislocation or subluxation was considered as a failure. Functional evaluation was based on the numeric pain scale, the Subjective Shoulder Value, and the Rowe Score and the Walch-Duplay Score.

### Radiological assessment

One independent observer (T. B.) used the open-source Horos DICOM 3-dimensional imaging software (DICOM, Danville, CA, USA) (GNU Lesser General Public License, version 3.0; Free Software Foundation, Boston, MA, USA) with a specific standardized protocol. Computed tomography (CT) scan was performed preoperatively, at 2 weeks and at a minimum of 2 years of follow-up postoperatively.

Preoperative CT scan evaluated bone loss according to Sugaya’s best fit circle method/diameter.^27^ The presence of Hill Sachs lesions was also recorded.

At 2 weeks postoperatively, CT scan evaluated graft positioning and glenoid width enlargement.

On the axial plane, the bone block was medial if the most lateral aspect was 2 mm medial to the glenoid rim, or lateral if it was 2 mm lateral to the glenoid rim. If it was in between, it was considered as flush. The contact surface between the bone block and the glenoid was measured according to Dalmas’ method.^9^

On the sagittal plane, the bone block was subequatorial if at least 50% of its height was below the glenoid equator. Glenoid width enlargement was determined by comparing preoperative best-fitting circle and postoperative new best-fitting circle diameters.^10^

At last follow-up, bone fusion was defined as trabeculation or ossified density crossing the glenoid/bone block space over a minimum length of 5 mm. Glenoid width enlargement and graft resorption was measured by comparing best-fitting circles between 2 weeks and the last CT scan.

Anteroposterior X-rays were performed at last follow-up, and signs of glenohumeral osteoarthritis were assessed according to the Samilson and Prieto classification.^24^

### Statistical analysis

Descriptive statistics (mean, range) and frequencies with proportions for categorical data were used to summarize recorded variables. Statistical analyses were performed using the EasyMedStat software (EasyMedStat, Levallois-Perret, France).

## Results

### Patient characteristics

Overall, 5 patients underwent this procedure, and none was lost to follow-up (Table I). There were 3 men and 2 women, and the mean age was 29 years. The dominant side was involved in 2 cases. Three patients were involved in recreational sport, 2 of whom played high risk activities (basketball, rugby). Three patients did light or heavy manual work. Three patients were regular smokers.

**Table I:** Patient characteristics.

Characteristic	n/5
Age	29 (20-52)
Gender	
Male	3
Female	2
Dominant side	2
Hyperlaxity, ER1 > 90°	1
Sport	
None	2
No risk	1
Overhead	0
Contact	0
Overhead and contact	2
Sport level	
None	2
Recreational	3
Competition	0
Work	
None	1
Nonmanual	1
Light manual	2
Heavy manual	1

*ER1*, external rotation at 0° abduction.

### Clinical results

The mean Index Severity Instability Score was 4.2 (range 3-5) (Table II).^3^ The preoperative active anterior elevation was 165° (range 140-170), the neutral external rotation was 62° (range 20-90), and external rotation at 90° was 95° (range 80-110). One patient with spondylarthritis had particularly low mobility, with 20° of neutral external rotation and 140° of anterior elevation. All patients reached interscapular internal rotation. Shoulder apprehension tests were positive preoperatively for all 5 patients.

**Table II:** Clinical assessment.

Clinical parameter	Preoperative	Last follow-up
Active ROM (mean, range)		
AE	165° (140-170)	170° (140-180)
ER1	62° (20-90)	57° (45-70)
ER2	95° (80-110)	85° (70-90)
IR	interscapular	interscapular
Functional scores (/100)		
SSV		89% (80-95)
Walch-Duplay		88 points (70-100)
Rowe		93 points (75-100)

*ROM*, range of motion; *AE*, anterior elevation; *ER1*, external rotation at 0° abduction; *ER2*, external rotation at 90° abduction; *IR*, internal rotation; *SSV*, Subjective Shoulder Value.

At mean follow-up of 50 months (range 44-56), none of our patients experienced recurrence of instability, neither dislocation nor subluxation. However, the Shoulder apprehension test was positive in one case.

Patients reported a mean Subjective Shoulder Value score of 89% (80-95). The mean Rowe Score was 93 points (75-100), and the mean Walch-Duplay Score was 88 points (70-100). All 5 patients were fully satisfied with the procedure.

### Radiological results

Preoperatively, the mean anterior glenoid bone loss was 13.8% (7-19) associated with a Hill Sachs lesion in all cases (Table III).

**Table III:** Radiological assessment as evaluated on preoperative and postoperative CT scan at 15 days and last follow-up.

Radiological assessment	Preoperative	Postoperative	Last follow-up
Bone defect			
Glenoid bone surface (%, range)	86.2 (82-93)	111 (100-128)	90 (82-108)
Hill Sachs lesion (n/5)	5		
Bone block positioning			
Vertical position			
Subequatorial		4	
Equatorial		0	
Supraequatorial		1	
Horizontal position			
Flush to glenoid rim		5	
Medial to glenoid rim > 2 mm		0	
Lateral to glenoid rim > 2 mm		0	
Contact surface (%, range)		59 (37-93)	
Resorption (%, range)			90 (50-100)
Osteoarthritis (n/5)			0

*CT*, computed tomography.

Postoperatively, bone block positioning was flush to the glenoid rim in all cases, and 4 were subequatorial (1 supraequatorial).

**Figure 1:**
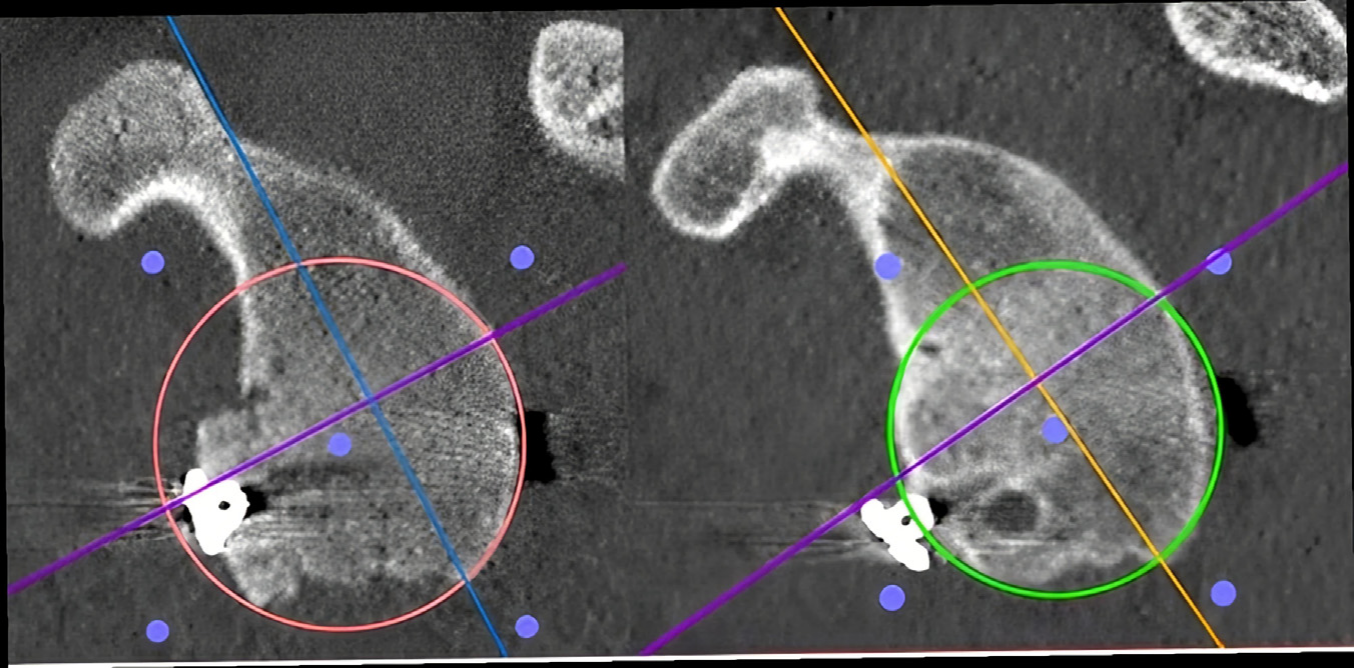
15 days postoperative (*left*) and last follow-up (*right*) CT scan evaluation. Glenoid en-face view showing the best-fit circle technique for bone reconstruction evaluation. *CT*, computed tomography.

**Figure 2:**
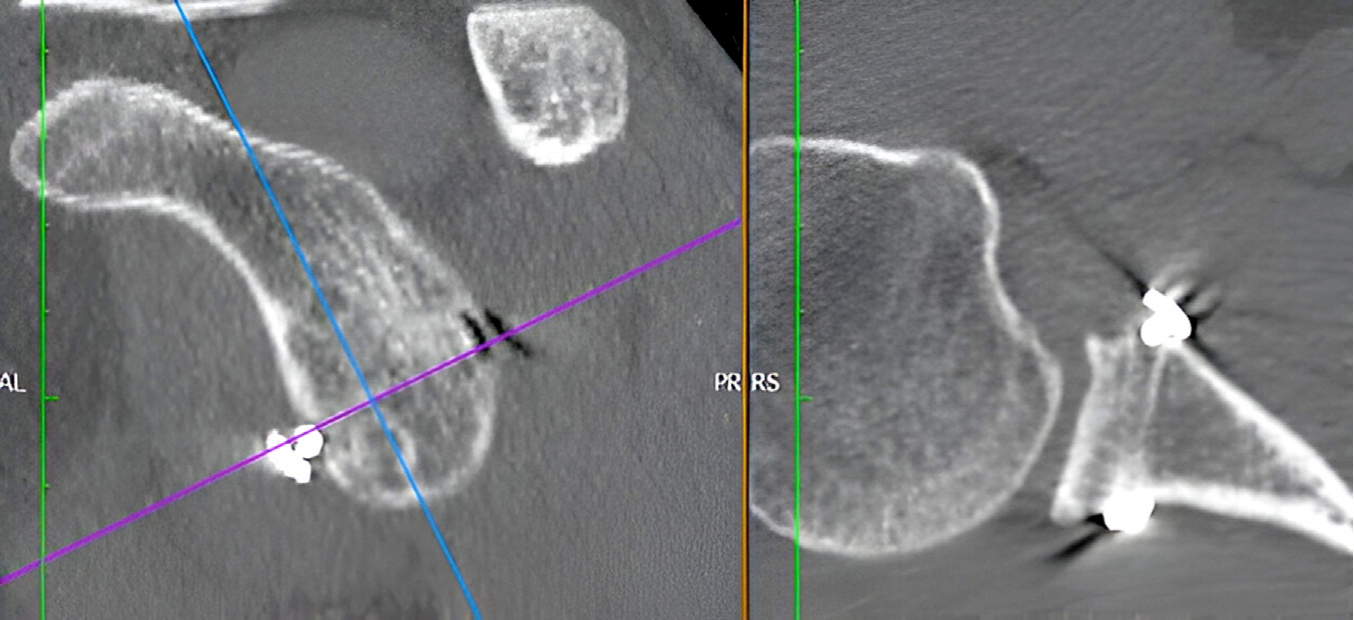
Last follow-up CT scan evaluation. Showing complete bone graft resorption and poor glenoid reconstruction. *CT*, computed tomography.

**Figure 3:**
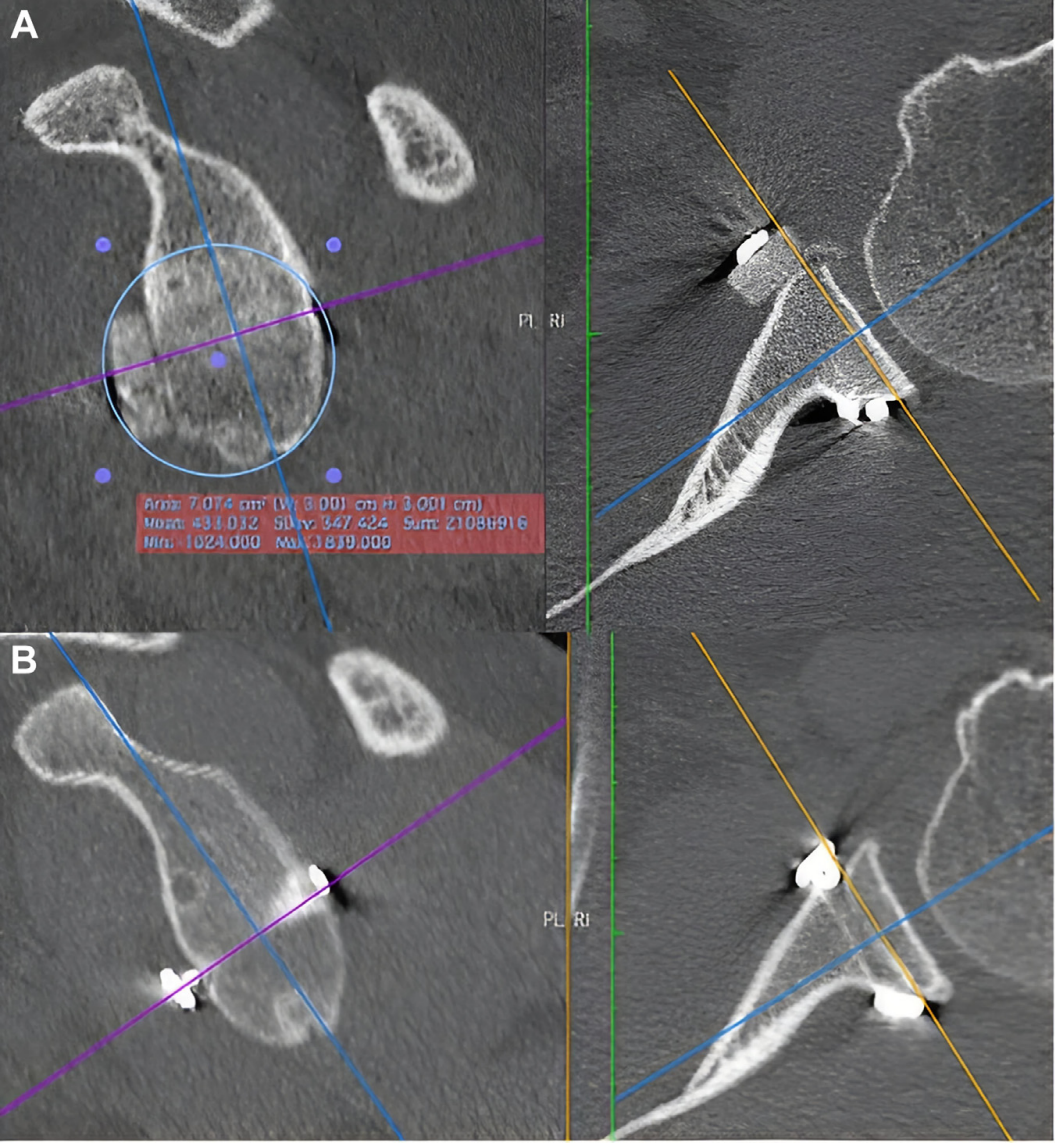
(**A**) 15 days postoperative and (**B**) last follow-up CT scan evaluation, showing bone graft complete resorption. *CT*, computed tomography.

Glenoid width enlarged from 86.2% to 111% at 2 weeks, and then decreased to 90% at last follow-up. Graft resorption occurred in all cases from 50% to 100% of the initial volume (Figure 1, Figure 2, Figure 3).

## Discussion

This study reported that arthroscopic Bankart repair augmented with free bone dry allograft in case of anterior shoulder instability with subcritical bone loss provided satisfactory clinical outcomes at mean follow-up of 50 months. However, major graft resorption occurred and therefore, it failed in compensating index glenoid bone loss.

Because recent studies demonstrated a high failure rate of isolated Bankart repair in case of glenoid bone defect, indications for bone block procedure increased with time.^5^^,^^18^^,^^26^ Besides critical bone loss (more than 25%) that formally requires glenoid reconstruction, recent studies described a gray zone called “subcritical” (from 13% to 17%) for which compensation seems to be controversial.^11^^,^^21^^,^^25^ An arthroscopic technique could provide the advantage of a minimal invasive procedure on the one hand, and a flexible dimensioned free bone graft to augment the Bankart repair on the other.

Using an allograft glenoid augmentation in primary surgery with significant glenoid bone defect or in case of revision after a failed Latarjet procedure has been common since the last decade. Provencher et al^22^ showed significant clinical improvement after glenoid augmentation using fresh distal tibia allograft in 27 patients. They reported no recurrence of instability at mean follow-up of 45 months and a resorption rate of only 3% (0%-25%). Franck et al^13^ compared 50 Latarjet procedures and 50 distal tibia allografts. They found no significant clinical difference (mean follow-up 45 months), with an overall recurrent instability rate of 1%. Each group had 5 complications and 3 reoperations.

The main issue of dry or fresh frozen allograft could be its poor integration and high rate of resorption. Boehm et al^4^ reported a series of 10 cases of revisions of anterior shoulder instability treated with a fresh frozen dry-preserved allograft. All allografts resorbed at 12 months of follow-up and an increase of glenoid bone loss was even identified. The allograft used in our study (Supercrit) is synthesized from human femoral heads obtained from hip replacement surgeries. The supercritical CO_2_ process allows virus inactivation without modifying the mineral and collagen content of the bone matrix. Trabecular tissue theoretically keeps its osteoconductive and mechanical properties equivalent to fresh bone.^14^ It has proven its safety and effectiveness in intervertebral fusion and dental surgery in particular.^2^^,^^7^ To our knowledge, this type of allograft has not been used for anterior shoulder instability yet. Unfortunately, in our study, 4 of 5 patients had complete resorption at follow-up of more than 4 years. The index glenoid bone loss was not compensated by using the allograft over time, leading to a recurrence of preoperative bone defect.

One explanation would be that, like other types of allografts, its biocompatibility remains unknown, inducing an immune-allergic reaction. The second explanation would be a technical issue since revascularization of the allograft requires sufficient contact area with native cancellous bone. In the case of glenoid augmentation, proper preparation of the anterior rim is mandatory. However, only one side can be in contact with host bone, which differs from using this material during reconstruction for replacement, where the allograft is impacted and compressed in a cavitary defect most of the time.^29^ Finally, even if we successfully oversized the glenoid to 111% of the primary glenoid area, it was previously reported that the less the glenoid bone loss, the greater the bone resorption.^12^ Our series included only patients with subcritical glenoid defect.

Despite the high resorption rate, none of the patients reported recurrence of instability. Rutger et al,^23^ in a systematic review, found 76% of partial to complete resorption among allograft glenoid augmentation, but no negative association with recurrence rate. Boehm et al^23^ reported only one case of apprehension following arthroscopic allograft reconstruction despite 100% resorption. Zhao et al^31^ described a bone remodeling process following a nonrigid fixation with anchors and reached a successful result in 94% of cases. Our findings are consistent with these results. All patients achieved excellent outcomes and high satisfaction, although it should be noted that none participated in high-risk activities for their shoulder.

The clinical outcomes, despite significant graft resorption, can be interpreted in several ways. First, complete resorption of the graft ultimately results in an isolated Bankart repair. In patients with subcritical bone loss and moderate physical activity, this technique might still hold value in the surgical orientation. Second, the presence of the graft may have provided mechanical protection during capsulolabral healing. It may have led ultimately to scar tissue formation in the anterior-inferior zone which could have a stabilization effect that enhances the properties of the Bankart repair.

Suture buttons have proven to be safe in the graft’s fixation, with low rate of reintervention and hardware failure.^6^^,^^20^ In the case of complete resorption, buttons might loosen from the scar tissue and cause intra-articular damage. Yet no migration was found in this study.

Sugaya’s linear method is known to overestimate glenoid bone loss. However, among all the techniques described to date, none can be considered the gold standard.^28^^,^^17^ In our study, the absolute value of the preoperative defect is only relevant in the initial diagnosis. The measurement technique we use is primarily designed to assess the relative variation of the inferior glenoid diameter over time and clearly illustrates graft resorption. Still, it is indeed essential to bear in mind the limitations of the method employed to correctly address patients.

This study has some weaknesses. It is a pilot evaluation with a small number of cases. Indeed, our first radiological results were so concerning that we stopped patient enrollment for this procedure despite satisfying clinical results. On the other hand, to our knowledge, this was the first study on bone dry allograft augmentation in anterior shoulder instability performed arthroscopically with a minimum follow-up of 2 years.

## Conclusion

This study has shown excellent clinical results of Bankart repair augmentation with free bone dry allograft in the treatment of anterior shoulder instability with glenoid subcritical bone loss. However, radiological evaluation demonstrated major resorption and poor glenoid bone reconstruction. Although this technique is attractive, it should not be recommended.

## Disclaimers:

Funding: No funding was disclosed by the authors.

Conflicts of interest: The authors, their immediate families, and any research foundation with which they are affiliated have not received any financial payments or other benefits from any commercial entity related to the subject of this article.

Given his role as Editor-in-Chief, Dr. Pierre Mansat had no involvement in the peer-review of this article and has no access to information regarding its peer-review. Full responsibility for the editorial process for this article was delegated to Dr. William J. Mallon.
